# Declining BMI and possible associations with changing perceptions of masculinity in Japanese young males

**DOI:** 10.1002/pcn5.70100

**Published:** 2025-04-09

**Authors:** Akihito Uezato, Saki Takagi, Yoshihiko Nakatani, Naoki Yamamoto

**Affiliations:** ^1^ Center for Basic Medical Research International University of Health and Welfare Tochigi Japan; ^2^ Center for Medical Innovation, Institute of Science Tokyo Tokyo Japan; ^3^ Department of Pharmaceutical Sciences International University of Health and Welfare Tochigi Japan

It is said that body image has been influenced by broader sociocultural trends. Previously, Kiriike and one of the authors reported the decreased body mass index (BMI) of young Japanese women from 1960 to 1995 and discussed the increased risk for eating disorders.^1^ In this report, we review changes in the BMI of young Japanese men and women over the past 20 years using publicly available data and discuss its possible association with shifts in perceptions of masculinity.

To represent the mid to late teens, the changes in the BMI of 17‐year‐old men and women from 2006 to 2024 are shown in Figure 1a. We chose 17‐year‐olds, as this is the oldest age for which data are available from the school health statistics in e‐Stat, the official portal site for Japanese Government Statistics (www.e-stat.go.jp/en). While the BMI of 17‐year‐old women remained relatively stable until 2019, a sharp decline was observed starting in 2020, during and even after the COVID‐19 pandemic. The BMI of 17‐year‐old men has continued to decrease gradually up to the present. Similarly, the BMIs of 15–19‐year‐old men and women showed comparable trends (Figures S1 and S2). Curiously, while the rate of underweight women remained stable until 2019, the rate among men steadily increased over the same period (Figure 1b).

**Figure 1:**
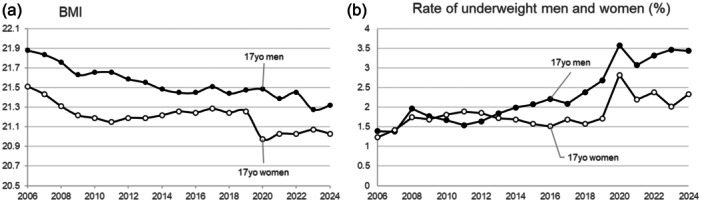
Changes in (a) body mass index (BMI) and (b) the rate of underweight 17‐year‐old men and women over the years from 2006 to 2024. Underweight is defined as a value equal to or less than −20% in the degree of obesity, calculated using the formula ([measured weight] − [standard weight by height])/[standard weight by height] × 100 (%).

It is unlikely that young people are eating less as the national health and nutrition survey shows the total energy intake of 15–19‐year‐old men and women appears to be constant in this period (Figure S3). Lifestyle changes, such as decreased physical activity due to increased smartphone usage and the subsequent decline in muscle mass, may be related to the reduction in BMI. However, according to a survey by the Japan Sports Agency, while grip strength among young people has shown a declining trend, core muscle strength has been increasing, making it difficult to draw clear conclusions about its relationship with BMI. Thus, although various factors may contribute to the decline in BMI, we aim to present the perspective that it is associated with changing perceptions of masculinity among Japanese people.

The perception of masculinity during the high economic growth in Japan, which ended in 1980s, was highly hegemonic focusing on productivity, labor, and breadwinning.^2^ During and after 1990s, the expectation of masculinities shifted from physical to “tender‐hearted” representation. A contemporary behavioral trend of young men, so‐called “Sohshokukei‐danshi,” herbivorous boys, has been a widely‐known buzzword for the past 15 years to indicate those who lack active association with women and prefer to engage in typically feminine acts such as cooking, eating sweets, or even making up.^3^ Their representation is in harmony with popular media icons such as idols produced by Japanese entertainment mega‐agencies, who are supposed to downplay any overt heteronormative masculinities and are not allowed to discuss any romantic relationship to maintain fans’ fantasy.^4^ The boom coincides with the BMI decline in young men (Figure 1). As “herbivorous boys” become increasingly accepted in Japanese society, reducing the pressure to conform to traditional masculinity, concerns about obesity continue to be raised through media messaging, which may ultimately contribute to a decline in BMI among boys. A 2016 study found that while 80% of male high school students fell within the normal BMI range (18.5–25), 30% desired weight loss.^5^ A study revealed that the rate of male students attempting weight reduction doubled in 2017 compared to 2010 (https://koueki.net/user/niye/110349438-2.pdf).

In Western society, concepts such as Men Going Their Own Way emphasize male autonomy but remain niche ideologies rather than widespread movements. Rather, the masculine ideal of lean muscularity and the resulting body dissatisfaction have remained consistent.^6^ Many western adolescent boys seek to gain weight—specifically by increasing muscle mass without becoming obese—a tendency that has been found to be associated with bulimic behavior.^7^, ^8^ While the phenomenon of “herbivorous boys” may seem unique to Japan or Asia, Japan has long been shaped by Western influence. Thus, the Western masculine ideal of lean muscularity may become more prominent. Conversely, as societal values diversify, the gentle, nonhegemonic masculinity of herbivorous boys could emerge as a global trend. Given the rising prevalence of eating disorders in men and concerns over distorted body image,^9^ sociocultural trends and masculinity‐related identity shifts remain key topics for future research. One promising approach is using tools like the Muscularity Oriented Eating Test to examine how young men are influenced by sociocultural factors.^10^

## AUTHOR CONTRIBUTIONS

*Data acquisition*: Saki Takagi and Yoshihiko Nakatani. *Data analysis*: Akihito Uezato and Saki Takagi. *Manuscript drafting*: Akihito Uezato and Naoki Yamamoto. All authors approved the final manuscript.

## CONFLICT OF INTEREST STATEMENT

The authors declare no conflict of interest.

## ETHICS APPROVAL STATEMENT

N/A

## PATIENT CONSENT STATEMENT

N/A

## CLINICAL TRIAL REGISTRATION

N/A

## Supporting information

Supporting information.

## Data Availability

The data used in this report were obtained from the publicly available official portal site for Japanese Government Statistics.
