# Supplementary material for: Declining BMI and possible associations with changing perceptions of masculinity in Japanese young males
Source: PCN Rep. 2025 Apr 9;4(2):e70100. doi: 10.1002/pcn5.70100 (PMC11982173; doi:10.1002/pcn5.70100)

**Supporting Information**

**Supplementary Figure 1.** Changes in BMI of 15-16-year-old men and women over the years from 2006 to 2024.


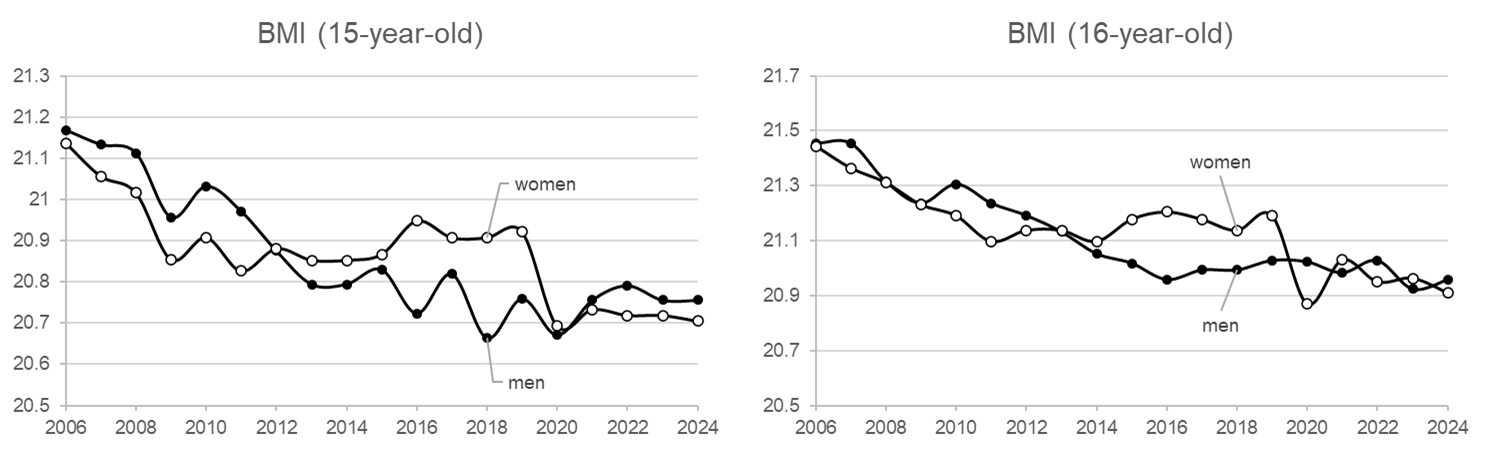


**Supplementary Figure 2.** Changes in BMI of 18-19-year-old men and women over the years from 2006 to 2023.


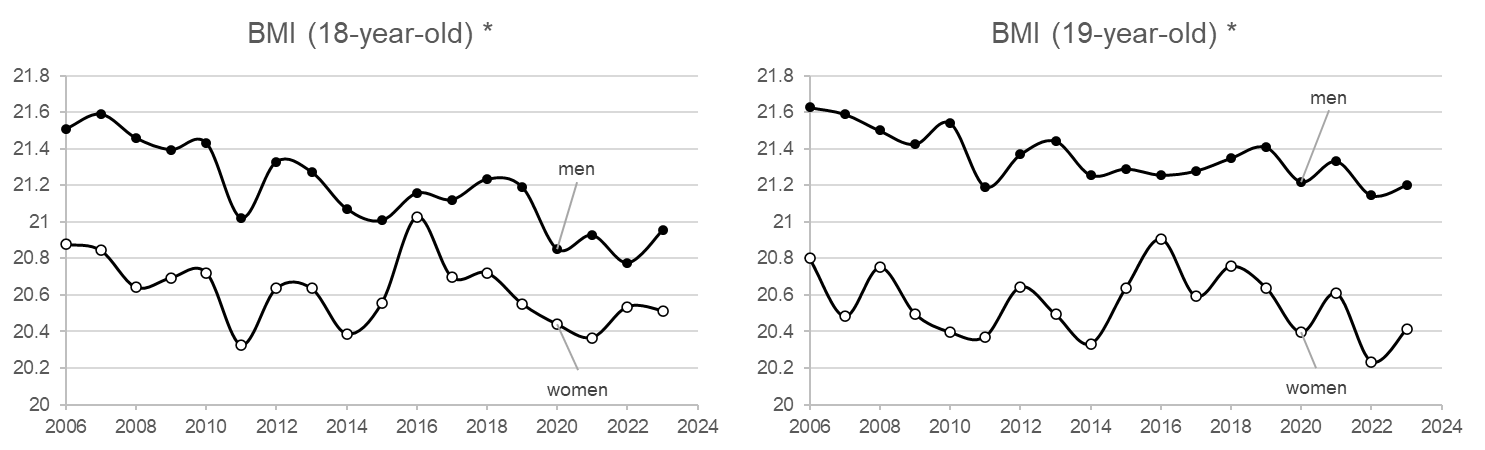


* The data was obtained from the dataset of the Physical Fitness and Athletic Ability Survey conducted by the Japan Sports Agency.

**Supplementary Figure 3.** Changes in total energy intake of 15-19-year-old men and women over the years from 2006 to 2023.


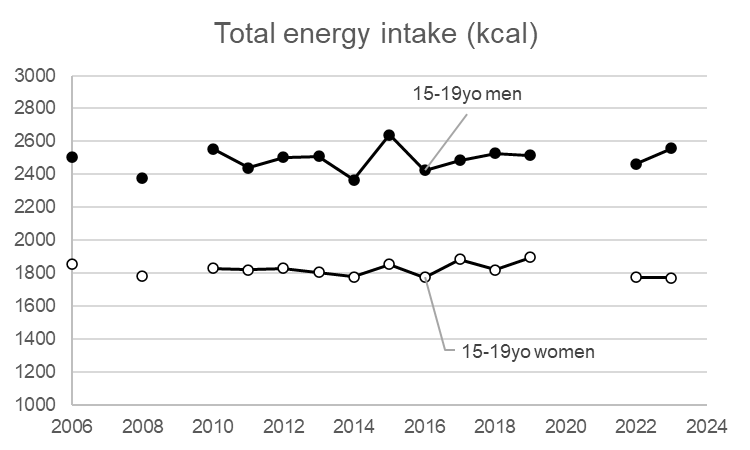

Supplement: Supplementary file 1 — Supporting information. [file PCN5-4-e70100-s001.docx]
